# Supplementary material for: Novel High-Entropy FeCoNiMoZn-Layered Hydroxide as an Efficient Electrocatalyst for the Oxygen Evolution Reaction
Source: Nanomaterials (Basel). 2024 May 20;14(10):889. doi: 10.3390/nano14100889 (PMC11123984; doi:10.3390/nano14100889)
Supplement: Supplementary file 1 [file nanomaterials-14-00889-s001.zip › nanomaterials-2987465-supplementary.pdf]

Supporting Information

Novel High-Entropy FeCoNiMoZn-Layered Hydroxide as an Efficient Electrocatalyst for the Oxygen Evolution Reaction

Zhihao Cheng [1], Xin Han [1], Liying Han [1], Jinfeng Zhang [1], Jie Liu [1,*], Zhong Wu [1] and Cheng Zhong [1,2]

[1] Key Laboratory of Advanced Ceramics and Machining Technology (Ministry of Education), and Tianjin Key Laboratory of Composite and Functional Materials, School of Materials Science and Engineering, Tianjin University, Tianjin 300072, China; 18002171015@163.com (Z.C.); 18822144032@163.com (X.H.); 18822029109@163.com (L.H.); jinfeng@tju.edu.cn (J.Z.); zhong.wu@tju.edu.cn (Z.W.); cheng.zhong@tju.edu.cn (C.Z.)
[2] Joint School of National University of Singapore and Tianjin University, International Campus of Tianjin University, Binhai New City, Fuzhou 350207, China
* Correspondence: jieliu0109@tju.edu.cn

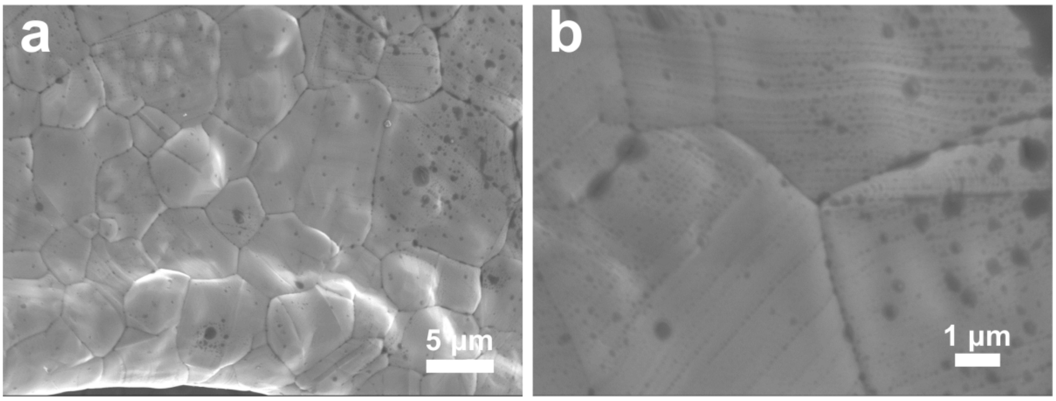

Figure S1. (a, b) SEM images of bare nickel foam.

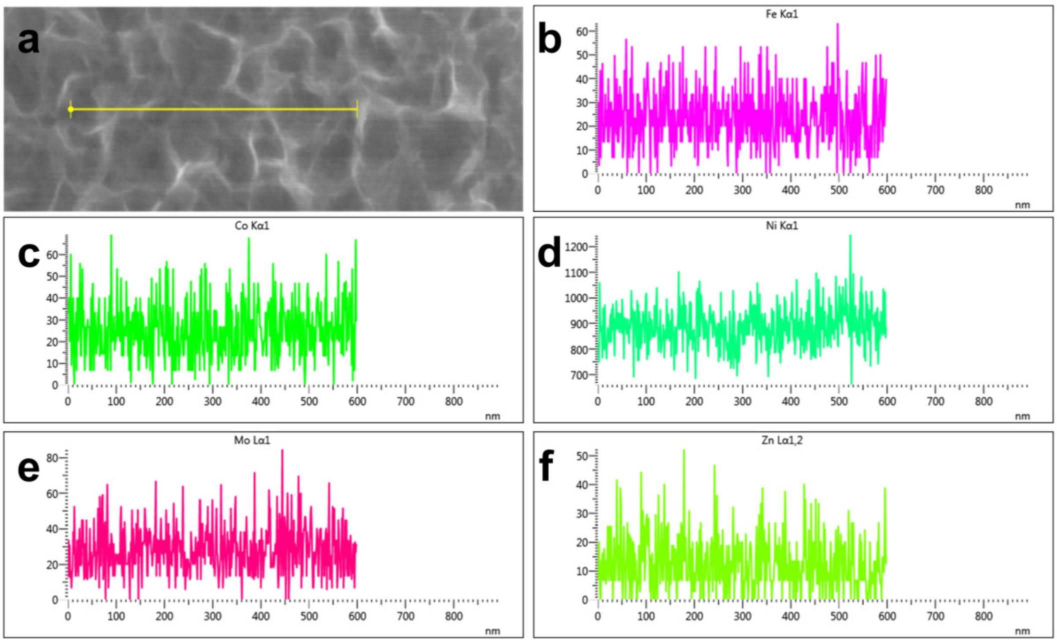

Figure S2. (a) SEM image, and line spectrum of FeCoNiMoZn-OH HEH, (b) Fe, (c) Co, (d) Ni, (e) Mo, (f) Zn.

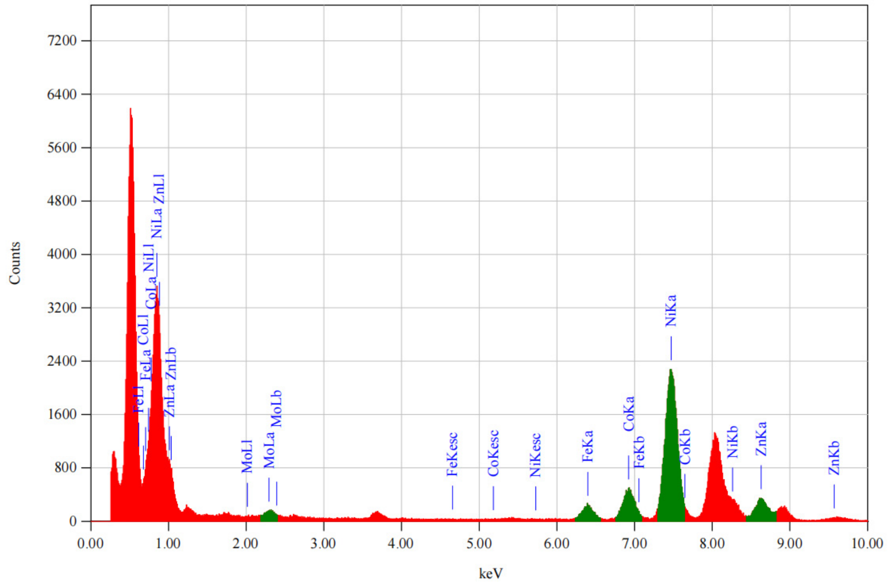

Figure S3. EDS spectrum of FeCoNiMoZn-OH HEH.

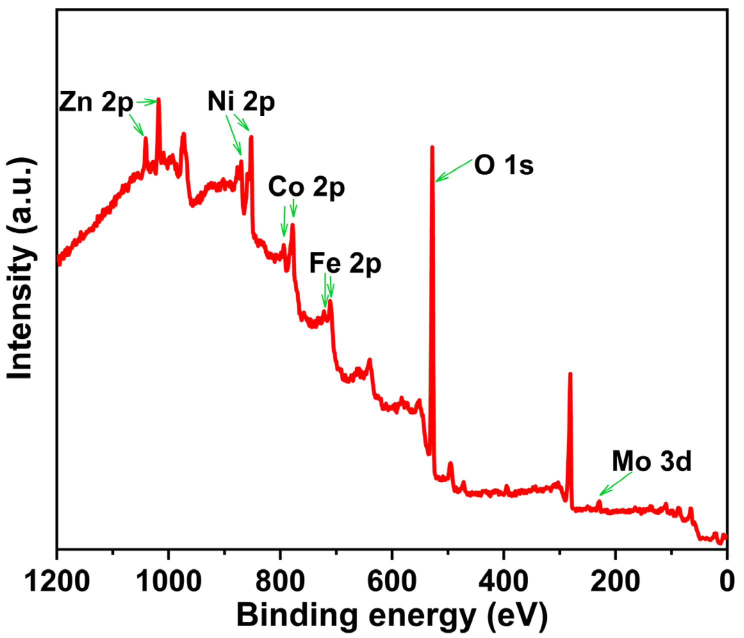

Figure S4. XPS survey spectrum of FeCoNiMoZn-OH sample.

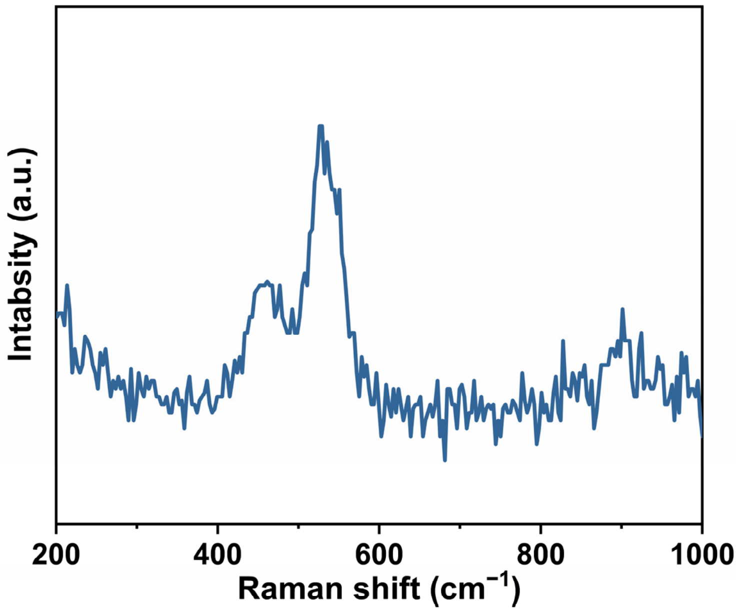

Figure S5. Raman spectrum of FeCoNiMoZn-OH sample.

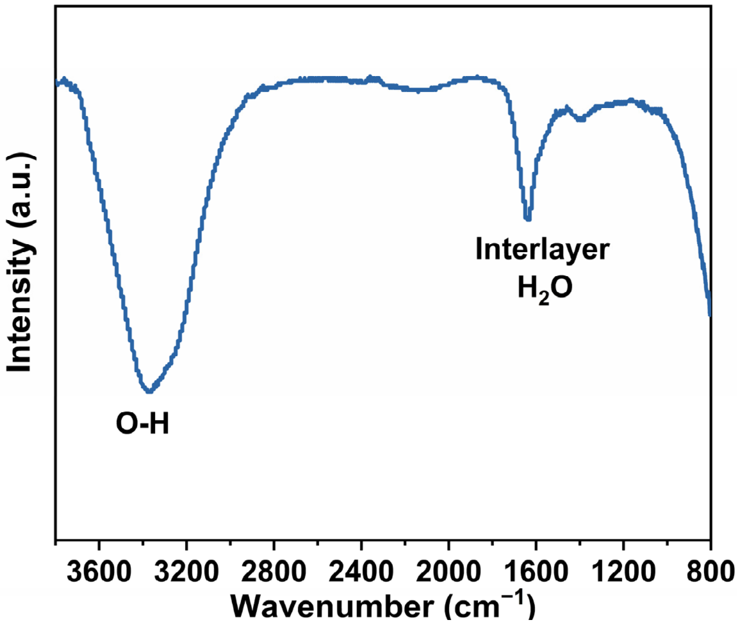

Figure S6. FTIR spectrum of FeCoNiMoZn-OH sample.

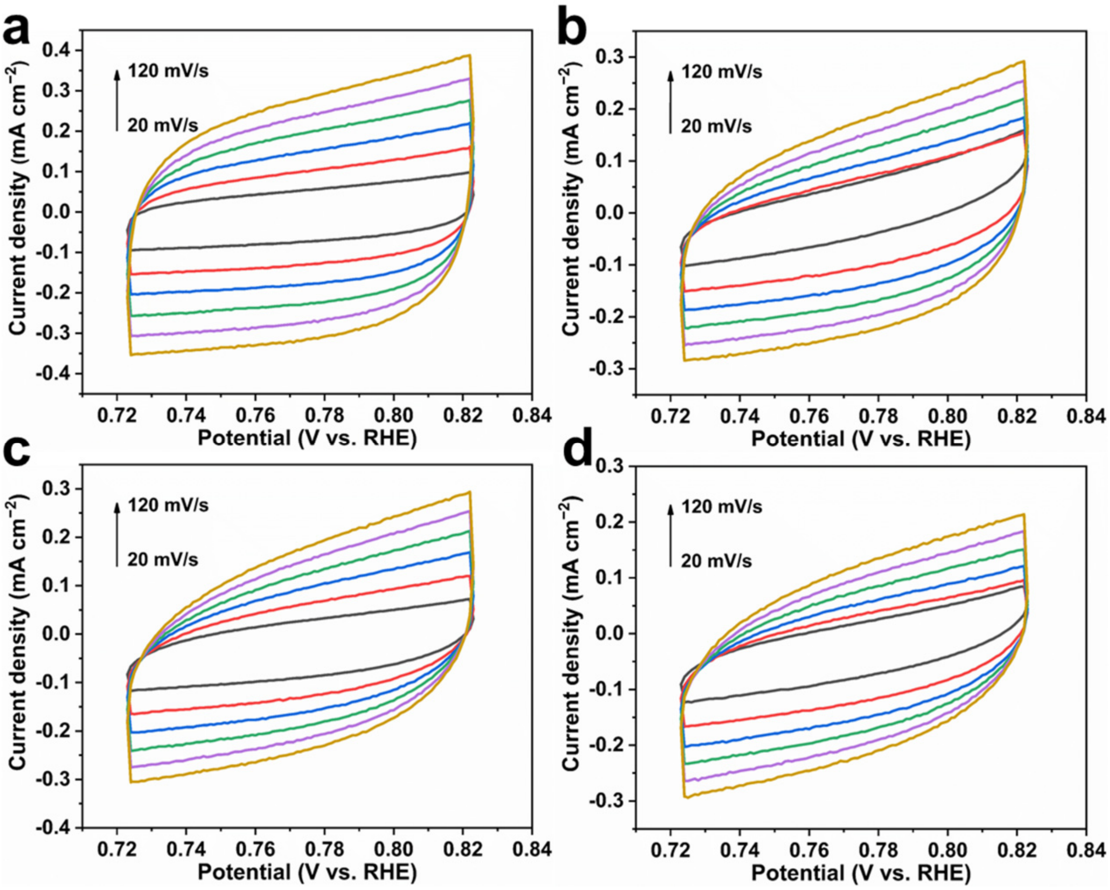

Figure S7. The CV curves of four samples at different scan rate (a) FeCoNiMoZn-OH, (b) FeCoNiMo-OH, (c) FeCoNiZn-OH, (d) FeCoNi-OH.

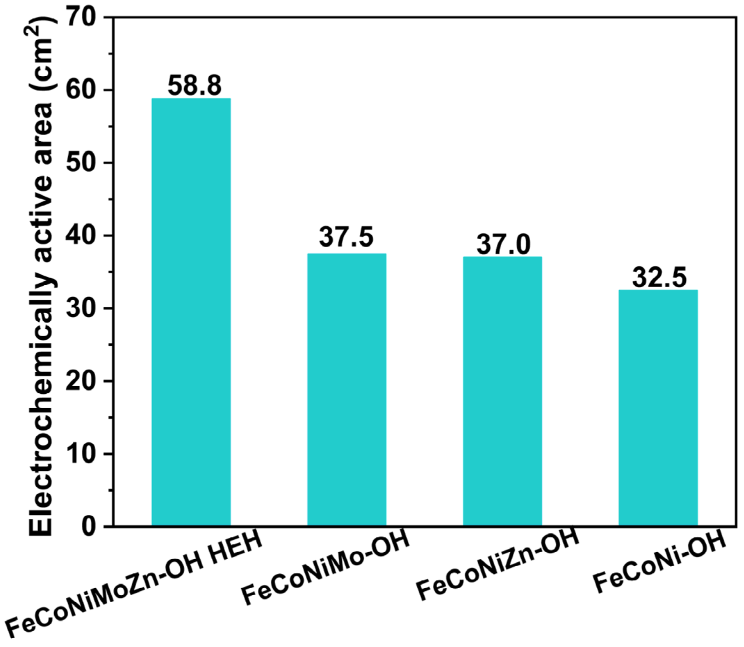

Figure S8. Histogram of electrochemically active area of four samples.

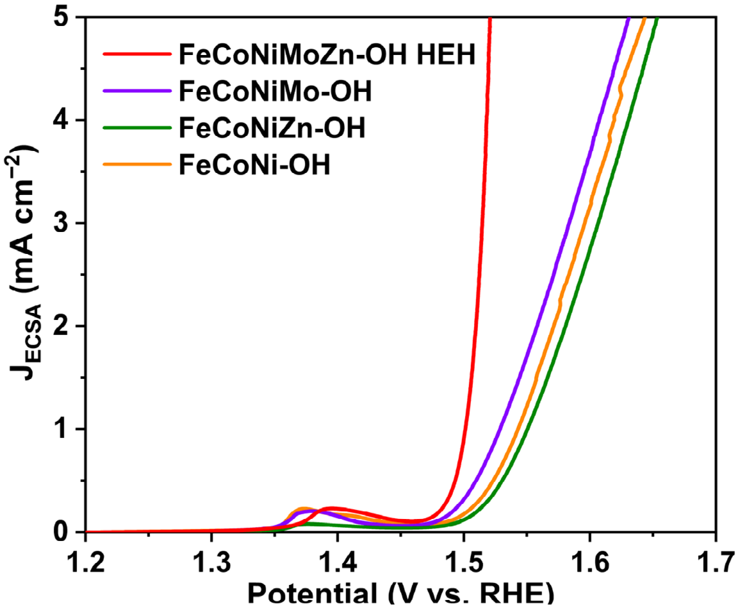

Figure S9. The LSV curves normalized by ECSA.

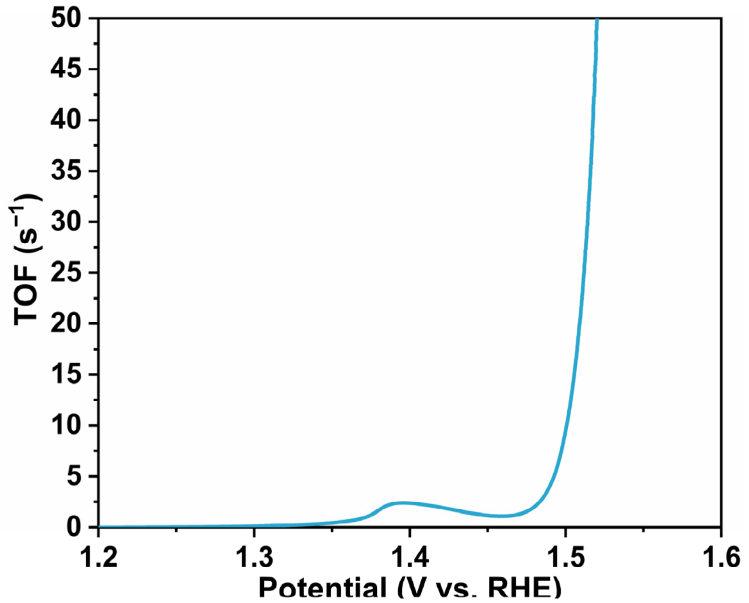

Figure S10. TOF curves of FeCoNiMoZn-OH HEH derived from LSV.

Table S1. Weight and atomic ratios of the corresponding elements of FeCoNiMoZn-OH HEH.

Elements	Mass %	Atom %
Fe	5.28	5.71
Co	13.17	13.51
Ni	65.24	67.2
Mo	5.09	3.21
Zn	11.21	10.36

Table S2. Comparison of TOF at the corresponding overpotentials of FeCoNiMoZn-OH HEH catalyst with recently reported other electrocatalysts.

Materials	Overpotential (mV)	TOF (s^{-1})	Ref.
NiIr-ENS	300	1.72	[1]
1D-CoP/3D-NiF	400	0.7	[2]
Ni − P_{24}	350	0.62	[3]
$FeCoNiCrMnO_x$	271	0.078	[4]
$Mg_{0.2}Co_{0.2}Ni_{0.2}Cu_{0.2}Zn_{0.2}O$	360	0.023	[5]
TA-Fe/Co-CNT	350	0.95	[6]
$Co_{0.75}Fe_{0.25}$@COF − TB	331	0.12	[7]
This work	250	1.81	-

References

1. Xie, Y.; Feng, Y.; Jin, S.; Li, C.; Li, C.; Sun, Y.; Luo, F., Yang, Z. Nickel-doped iridium echinus-like nanosheets for stable acidic water splitting. *Chem. Commun. (Cambridge, U. K.)* **2023**, 59, 8404-8407. 10.1039/d3cc02299d
2. Khiarak, B. N.; Zahraei, A. A.; Nazarzade, K.; Hasanjani, H. R. A., Mohammadzadeh, H. Shape-controlled synthesis of thorn-like 1D phosphorized Co supported by Ni foam electrocatalysts for overall water splitting. *J. Mater. Sci.-Mater. Electron.* **2021**, 32, 18363-18370. 10.1007/s10854-021-06379-3
3. Battiato, S.; Urso, M.; Cosentino, S.; Pellegrino, A. L.; Mirabella, S., Terrasi, A. Optimization of Oxygen Evolution Reaction with Electroless Deposited Ni-P Catalytic Nanocoating. *Nanomaterials* **2021**, 11, 3010. 10.3390/nano11113010
4. Shen, K.; Wang, T.; Li, C.; Chen, M.; Niu, L., Gong, Y. Designing highly-efficient oxygen evolution reaction FeCoNiCrMnOx electrocatalyst via coexisted crystalline and amorphous Phases: Experiment and theory. *Appl. Surf. Sci.* **2024**, 650, 159102. 10.1016/j.apsusc.2023.159102
5. Liu, F.; Yu, M.; Chen, X.; Li, J.; Liu, H., Cheng, F. Defective high-entropy rocksalt oxide with enhanced metal-oxygen covalency for electrocatalytic oxygen evolution. *Chin. J. Catal.* **2022**, 43, 122-129. 10.1016/s1872-2067(21)63794-4
6. Zhu, W.; Wang, X.; Zhu, Y.; Fang, L.; Yao, C.; Song, X.; Chen, H.; Wang, X., Zhu, G. Amorphous Fe/Co-based tannic acid salts as robust oxygen evolution pre-catalysts. *New J. Chem.* **2023**, 47, 19087-19095. 10.1039/d3nj03258b
7. Liu, X.; Feng, L.; Li, Y.; Xia, T.; Sui, Z., Chen, Q. Covalent Organic Frameworks Composites Containing Bipyridine Metal Complex for Oxygen Evolution and Methane Conversion. *Molecules* **2022**, 27, 5193. 10.3390/molecules27165193
